# Efficacy of central extracorporeal life support for patients with fulminant myocarditis and cardiogenic shock

**DOI:** 10.1093/ejcts/ezab231

**Published:** 2021-06-25

**Authors:** Naoki Tadokoro, Satsuki Fukushima, Kimito Minami, Takura Taguchi, Tetsuya Saito, Naonori Kawamoto, Takashi Kakuta, Osamu Seguchi, Takuya Watanabe, Seiko Nakajima Doi, Kensuke Kuroda, Keisuke Suzuki, Masanobu Yanase, Yasuhide Asaumi, Hideyuki Shimizu, Norihide Fukushima, Tomoyuki Fujita

**Affiliations:** 1 Department of Cardiovascular Surgery, National Cerebral and Cardiovascular Center, Suita, Osaka, Japan; 2 Department of Cardiovascular Surgery, Keio University School of Medicine, Shinjuku-ku, Tokyo, Japan; 3 Department of Surgical Intensive Care, National Cerebral and Cardiovascular Center, Suita, Osaka, Japan; 4 Department of Transplantation, National Cerebral and Cardiovascular Center, Suita, Osaka, Japan; 5 Department of Cardiovascular Medicine, National Cerebral and Cardiovascular Center, Suita, Osaka, Japan

**Keywords:** Fulminant myocarditis, Mechanical circulatory support, Extracorporeal life support, Ventricular assist device, Creatine kinase-MB, Rhythm disturbance

## Abstract

**OBJECTIVES:**

Fulminant myocarditis with cardiogenic shock requires extracorporeal life support (ECLS) and has poor outcomes. To improve outcomes, we have converted patients with severely impaired cardiac and multiorgan function from peripheral to central ECLS. In this study, we reviewed these patients’ clinical outcomes and investigated associated factors.

**METHODS:**

We retrospectively studied 70 consecutive patients with fulminant myocarditis under peripheral support from 2006 to 2020. Forty-eight patients underwent surgical conversion to central support, and the remaining patients continued peripheral support. The end point was survival and ventricular assist device-free survival.

**RESULTS:**

More severe pulmonary congestion and multiorgan failure were present in patients with central than peripheral support. Weaning from ECLS was achieved in 95% and 62% of patients with peripheral and central support, respectively. Five-year survival was not significantly different between patients with central and peripheral support (71.2% vs 87.5%, respectively; *P* = 0.15). However, the ventricular assist device-free survival rate was significantly higher in patients with central than peripheral support (82.2% vs 52.0%, respectively; *P* = 0.017). A peak creatine kinase-MB level of >180 IU/l, rhythm disturbance and aortic valve closure were detrimental to functional recovery in patients with central support.

**CONCLUSIONS:**

Conversion to central ECLS is feasible and safe in patients with fulminant myocarditis. Patients with severe myocardial injury as shown by a high creatine kinase-MB level, rhythm disturbance and aortic valve closure should be converted to a durable left ventricular assist device.

**Figure:**
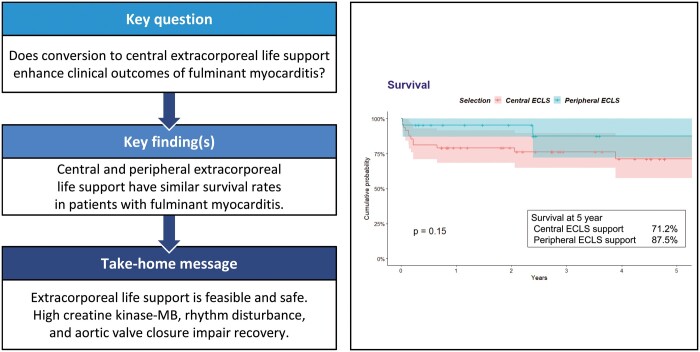

## INTRODUCTION

Viral infection can trigger myocardial inflammation, leading to the deterioration of cardiac function and congestive heart failure. Patients with progressive haemodynamic deterioration despite optimum medical therapy require extracorporeal life support (ECLS) to maintain end-organ circulation until cardiac function recovers. This critical state of acute myocarditis is called fulminant myocarditis (FM) [1, 2]. Generally, ECLS for FM is the first established by peripheral arterial and venous cannulations. Although this peripheral ECLS is promptly established even at the bedside, it is often complicated by insufficient flow support, lung oedema or complications such as bleeding or limb ischaemia in patients with poor cardiac function. In addition, patients without improvement in cardiac function within 48 h after introduction of peripheral ECLS have a poor prognosis and require further treatment [3]. We recently reported the safety and therapeutic efficacy of conversion from peripheral to central ECLS [4], which maximizes end-organ circulation and ventricular unloading in patients with refractory congestive heart failure of any cause. In particular, combination of an extracorporeal ventricular assist device (VAD) and extracorporeal membrane oxygenation (ECMO) by central cannulations contributes to the salvage of patients with a critical haemodynamic state, even under peripheral ECLS.

Patients with FM show a markedly variable time course of functional changes and degree of functional prognosis. Although the factors that determine this variability are incompletely understood, we consider that left ventricular (LV) wall stress may affect the magnitude of myocardial inflammation and consequently determine the functional prognosis. Our hypothesis was that conversion to central ECLS is feasible and safe for patients under suboptimum and/or prolonged peripheral ECLS. Therefore, we reviewed the outcomes of patients with FM under peripheral and/or central ECLS and verified our institutional strategy of conversion to central ECLS.

## MATERIALS AND METHODS

### Study cohort and data collection

This retrospective, single-centre study involved 70 consecutive patients who had FM-related refractory congestive heart failure and were treated by peripheral ECLS in the National Cerebral and Cardiovascular Centre from February 2006 to July 2020. FM was diagnosed by pathological findings with additional anatomical or haemodynamic findings, as documented in the Supplementary Material. Study data were collected by reviewing the patients’ medical charts, surgical reports and referral letters. Follow-up was completed at the study conclusion in all patients. Data collection was performed in October 2020. All patients or their legal representatives provided written informed consent for surgery and use of their data for diagnostic and research purposes. This study was approved by the National Cerebral and Cardiovascular Center review board (approval number: M30-026, approval date: 18 July 2018).

### Indication for conversion to central extracorporeal life support

Prior to introduction of the Impella pump catheter program, conversion to central ECLS was determined when multiorgan failure was exacerbated or cardiac function did not recover for >2 days, even with optimum peripheral ECLS. After launching of the Impella in January 2018, peripheral ECLS was optimized by initially adding the Impella if patients had adequate cardiac anatomy and vascular access for insertion of the Impella. Throughout this study, the core concept of ECLS for myocarditis was conversion to central support in patients with suboptimum and/or prolonged peripheral ECLS, including the Impella.

Indications for conversion from peripheral to central ECLS and the modality of central ECLS were intensively discussed.

**Figure 1:**
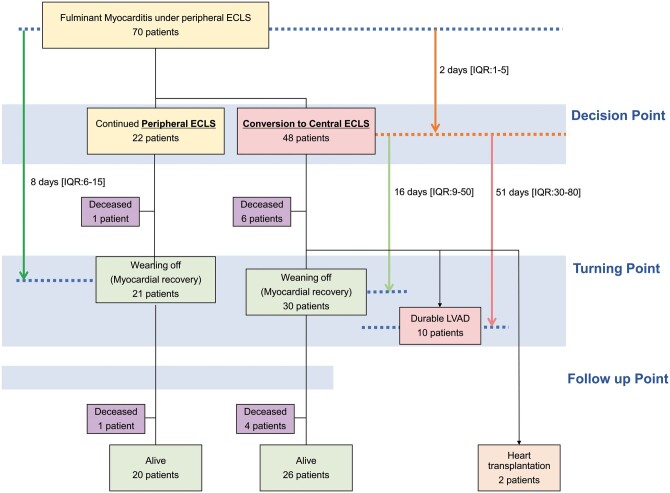
Schema of clinical outcomes of study cohort. ECLS: extracorporeal life support; IQR: interquartile range; LVAD: left ventricular assist device.

**Figure 2:**
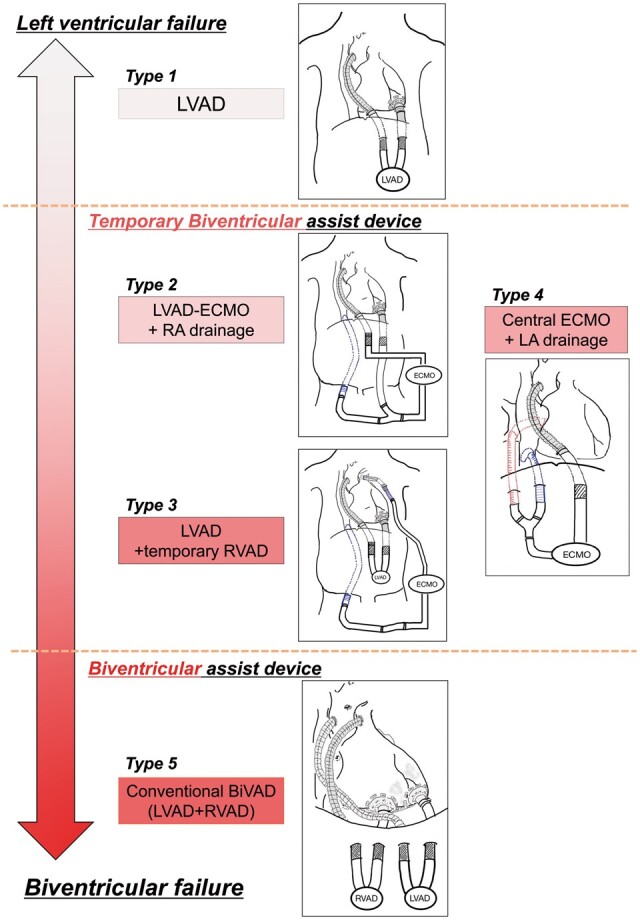
Types and selection of central ventricular assist device (extracorporeal life support). BiVAD: biventricular ventricular assist device; ECMO: extracorporeal membrane oxygenation; LA: Left atrium; LVAD: left ventricular assist device; RA: right atrium; RVAD: right ventricular assist device.

Central ECLS was defined as an ECLS system in which an outflow cannula was placed in the ascending aorta and inflow cannulas were placed in the left ventricle, right ventricle and/or right atrium under median sternotomy. Peripheral ECLS was defined as the placement of an intra-aortic balloon pump (IABP), venoarterial (VA)-ECMO and/or Impella pump catheter by peripheral cannulation. All 70 patients underwent peripheral ECLS upon enrolment. Forty-eight patients subsequently underwent conversion to central ECLS and the remaining 22 continued peripheral ECLS (Fig. 1). The standard modality of central ECLS was a left ventricular assist device (LVAD); however, FM is often associated with right ventricular (RV) failure and lung oedema, which require a right VAD and/or ECMO (Fig. 2). In most patients, we first placed an LVAD to assess whether it provided sufficient flow support. According to the LVAD flow and haemodynamics, we then considered the need for additional RV and/or lung support. When the LVAD flow was ≤3.0 l/min/m^2^, we added either right atrial drainage (type 2) or temporary RV support (type 3). Type 3 was selected when the pulmonary artery pulse pressure/right atrial pressure ratio (pulmonary artery pulsatility index) was <1.8; otherwise, type 2 was selected [5]. In patients with severe heart, lung, and systemic oedema, type 4 (establishment of central ECMO by left and right atrial drainage) was selected to avoid cannulation to a fragile and small left ventricle [4]. Type 5 was selected in patients with critical LV/RV dysfunction, such as asystole and without severe lung/heart oedema, to establish stable biventricular support without the need for redo surgeries until implantation of a durable device.

### Protocol of weaning from extracorporeal life support

Weaning from peripheral ECLS, such as VA-ECMO or the Impella 5.0, was performed as described previously [6, 7]. Conversely, weaning from central ECLS was based on the Berlin criteria or other conditions as described in the Supplementary Material [8]. Patients who failed weaning from central ECLS subsequently underwent implantation of a durable LVAD as a bridge to transplantation.

### Evaluation of timing of echocardiography, laboratory data and electrocardiography

All patients were examined at least 3 times (Fig. 1): (i) immediately before conversion to central ECLS or 2–4 days after introducing peripheral ECLS in the peripheral ECLS group (decision point) because the median duration from starting peripheral ECLS to central ECLS conversion was 2 days, (ii) immediately before weaning from ECLS or conversion surgery for a durable LVAD in surviving patients (turning point) and (iii) within 2 months after weaning in patients who were successfully weaned from peripheral/central ECLS (follow-up point).

Cardiac function was estimated by echocardiographic and electrocardiographic findings and right heart catheterization. The degree of multiorgan failure was evaluated by the Model of End-stage Liver Disease score, Kidney Disease Improving Global Outcomes classification and lung oedema grade. The primary end point was survival and VAD-free survival.

### Endomyocardial biopsy

Endomyocardial biopsy was performed from the RV wall or apical portion of the left ventricle in patients who underwent central ECLS implant surgery. These specimens were examined to determine the presence of myocarditis or borderline myocarditis according to the Dallas pathological criteria (Supplementary Material) [9].

### Statistical analyses

Continuous variables are shown as median and interquartile range (IQR). Categorical and ordinal variables are shown as number and percentage. The cumulative probability of survival and VAD-free survival were computed using the Kaplan–Meier method. The log-rank test was used to compare survival and VAD-free survival between the central and peripheral ECLS groups.

To assess the effects of risk factors for non-myocardial recovery among patients undergoing central ECLS, a univariable logistic regression model using the central ECLS cohort data was fitted with the following 9 factors as independent variables: age, body surface area, male sex, rhythm disturbance [asystole or complete atrioventricular block (CAVB)], peak creatine kinase (CK)-MB level of >180 IU/ml, aortic valve closure at central ECLS conversion (decision point), requirement for biventricular support, interval from initiation of peripheral ECLS to central ECLS conversion and interval of biventricular support. These variables were chosen because they might be associated with the type of central ECLS and non-myocardial recovery. In particular, the CK-MB level was selected based on our previous institutional reports [6]. To assess the effects of risk factors for non-myocardial recovery, multivariable logistic regression was fitted using age, sex and any variable with a *P*-value of ≤0.05 in the previous univariate analysis. Age and sex were used in the multivariable analysis because they are common confounders for death in the general population.

Missing data were imputed with the multiple imputation method using the ‘aregImpute’ function of the rms package in R. Missing data for all independent variables used in the statistical model were imputed 5 times. No statistical power calculations were performed before the study. The sample size was based on data availability. Statistical analyses were performed with a two-sided 5% significance level using R 3.6.0 (The R Foundation for Statistical Computing, Vienna, Austria).

## RESULTS

### Patients’ characteristics and selection of extracorporeal life support

Among 70 patients diagnosed with FM in our centre, 46 (66%) underwent peripheral ECLS at a referral hospital. We subsequently performed conversion surgery from peripheral to central ECLS in 48 (69%) patients. Among the patients with central ECLS, 33 (68%) required biventricular support at the decision point. The median interval from peripheral ECLS initiation to central ECLS conversion was 2 days (IQR 1–5).

Conversion to central ECLS was performed in patients who received suboptimal peripheral ECLS. Therefore, the patients’ backgrounds and characteristics differed greatly between the central and peripheral ECLS groups at the decision point (Table 1). The peak CK, peak CK-MB and serum creatinine levels and the international normalized ratio were higher in the central than peripheral ECLS group. The LV ejection fraction and aortic opening time were lower in the central than peripheral ECLS group. The Model of End-stage Liver Disease score, Kidney Disease Improving Global Outcomes classification and lung oedema grade were also worse in the central than peripheral ECLS group.

**Table 1:** Patients characteristics in total cohort and each support group

	Central ECLS (*n* = 48)	Peripheral ECLS (*n* = 22)
Characteristics		
Age (years)	44 (33–58)	50 (42–69)
Body surface area (m^2^)	1.64 (1.55–1.77)	1.68 (1.58–1.84)
Male gender	26 (54.2)	14 (63.6)
Symptoms		
Fever >38.0°C	34 (70.8)	15 (68.2)
Chest pain	16 (33.3)	5 (22.7)
Digestive symptoms	9 (18.8)	3 (13.6)
Respiratory symptoms	15 (31.2)	12 (54.5)
Interval between symptom onset and initiation of peripheral ECLS	4 (3–7)	5 (4–7)
Pathology		
Lymphocytic	39 (81.2)	14 (63.6)
Eosinophilic	1 (2.1)	5 (22.7)
Giant cell	4 (8.3)	1 (4.5)
Borderline	4 (8.3)	2 (9.1)
Peripheral ECLS selection immediately before decision point		
IABP	44 (91.7)	21 (95.5)
VA-ECMO	44 (91.7)	18 (81.8)
Impella	5 (10.4)	8 (36.4)
Impella 2.5	1 (20.0)	1 (12.5)
ImpellaCP	3 (60.0)	0 (0.0)
Impella 5.0	1 (20.0)	7 (87.5)
Medical treatment		
Intravenous immunoglobulin	45 (93.8)	12 (54.5)
Glucocorticoid treatment	31 (64.6)	11 (50.0)
Laboratory data		
Peak CK (IU/l)	2952 (1665–6552)	1249 (518–2846)
Peak CK-MB (IU/l)	144 (66–324)	59 (32–134)
Serum creatinine (mg/dl)	1.25 (0.80–2.25)	0.70 (0.62–1.25)
AST (IU/l)	356 (171–928)	187 (45–564)
ALT (IU/l)	177 (92–563)	97 (49–778)
Lactate dehydrogenase (IU/l)	1354 (1067–2212)	753 (475–1440)
Prothrombin time-international normalized ratio	1.22 (1.15–1.57)	1.16 (1.02–1.25)
Multiorgan function		
MELD score	20 (9–27)	9 (6–19)
KDIGO		
Stage 1	17 (35.4)	15 (68.2)
Stage 2	5 (10.4)	1 (4.5)
Stage 3	26 (54.2)	6 (27.3)
Continuous renal replacement therapy	25 (52.1)	6 (27.3)
Pulmonary congestion grading		
Grade 1	12 (25.0)	16 (72.7)
Grade 2	14 (29.2)	4 (18.2)
Grade 3	22 (45.8)	2 (9.1)
Electrocardiographic disturbance		
Complete atrioventricular block	27 (56.2)	13 (59.1)
Transient complete atrioventricular block	22 (81.5)	12 (92.3)
Asystole	7 (14.6)	1 (4.5)
Transient asystole	5 (71.4)	1 (100.0)
Transthoracic echocardiography		
LVEF (%)	8.5 (6.2–12.1)	24.4 (14.0–32.9)
LVDd (mm)	47.0 (40.7–52.0)	47.0 (43.0–55.3)
LVDs (mm)	45.0 (36.7–48.2)	40.0 (37.0–46.0)
Closed aortic valve	27 (56.2)	2 (9.1)

Data are presented as median (interquartile range) or *n* (%).

ALT: alanine aminotransferase; AST: aspartate aminotransferase; CK: creatine kinase; ECLS: extracorporeal life support; IABP: intra-aortic balloon pump; LVDd: left ventricular internal diameter in diastole; LVDs: left ventricular internal diameter in systole; LVEF: left ventricular ejection fraction; MELD: Model of End-stage Liver Disease; KDIGO: Kidney Disease Improving Global Outcomes; VA-ECMO: venoarterial extracorporeal membrane oxygenation.

### Early outcomes from starting point to turning point

One patent with peripheral ECLS died of sudden retroperitoneal bleeding. The remaining 21 patients reached the turning point and were weaned from peripheral ECLS with a median support duration of 8 days (IQR 6–15) (Table 2).

**Table 2:** Characteristics of weaning versus non-weaning in patients with central ECLS

	Weaning from central ECLS (*n* = 30)	Non-weaning from central ECLS (*n* = 18)
Preoperative characteristics		
Age (years)	50 (37–58)	42 (27–52)
Body surface area (m^2^)	1.65 (1.54–1.77)	1.69 (1.57–1.77)
Male gender	14 (46.7)	12 (66.7)
Pathology		
Lymphocytic myocarditis	23 (76.7)	16 (88.9)
Others	7 (23.3)	2 (11.2)
Laboratory data		
Peak CK	2111 (945–5000)	5672 (2830–9657)
Peak CK-MB	120 (51–147)	295 (148–516)
MELD score	20 (6–26)	21 (15–27)
Electrocardiographic disturbance		
CAVB	12 (40.0)	15 (83.3)
Asystole	1 (3.3)	6 (33.3)
Central ECLS primary systems		
LVAD	10 (33.3)	5 (27.8)
Biventricular support	20 (66.6)	13 (82.2)
Time course		
Central ECLS (days)	16 (9–50)	55 (31–134)
Interval of biventricular support (days)	6 (4–7)	22 (8–79)
Transthoracic echocardiography at decision point		
LVDd (mm)	48.0 (40.2–54.0)	46.0 (41.0–48.5)
LVDs (mm)	45.0 (36.2–49.0)	45.0 (38.5–46.5)
LVEF (%)	11.0 (7.3–14.4)	7.6 (5.7–8.4)
Closed aortic valve	13 (43.3)	15 (82.4)

Data are presented as median (interquartile range) or *n* (%).

CAVB: complete atrioventricular block; CK: creatine kinase; ECLS: extracorporeal life support; LVAD: left ventricular assist device; LVDd: left ventricular internal diameter in diastole; LVDs: left ventricular internal diameter in systole; LVEF: left ventricular ejection fraction; MELD: Model of End-stage Liver Disease.

Six patents with central ECLS died (LVAD, *n* = 2; biventricular assist device, *n* = 4). The causes of death were multiorgan failure (*n* = 2), thrombogenic cerebral infarction (*n* = 2), septicaemia (*n* = 1) and idiopathic interstitial pneumonia (*n* = 1). Complications among patients with central ECLS included cerebral infarction in 2 (3.5%), subarachnoid haemorrhage in 1 (1.7%) and deep sternal wound infection in 3 (5.3%). Early outcomes of the upgrade or downgrade strategy of the different types of central ECLS are described in detail in the Supplementary Material. Among the 42 surviving patients, 40 reached the turning point. The final support type at the turning point was an LVAD in 35 (72.9%) patients, biventricular assist device in 9 (18.8%), central ECMO in 3 (6.2%) and right VAD in 1 (2.1%). Additional details on system changes of the central ECLS procedure are described in the Supplementary Material.

Central ECLS was removed for weaning (*n* = 30) or conversion for durable LVAD implantation (*n* = 10). The median duration from central ECLS implantation (decision point) to weaning and durable LVAD conversion (turning point) was 16 days (IQR 9–50) and 54 days (IQR 30–80), respectively. The median duration of biventricular support was 7 days (IQR 5–28). In addition, 2 patients underwent heart transplantation under extracorporeal LVAD support at 374 and 968 days from the decision point. Rates of in-hospital death or major complications associated with post-conversion surgery were not significantly different among the support systems. Transthoracic echocardiography showed no significant differences between the 2 groups at the turning and follow-up points (Supplementary Material, Table S1).

### Survival and cardiac outcomes

**Figure 3:**
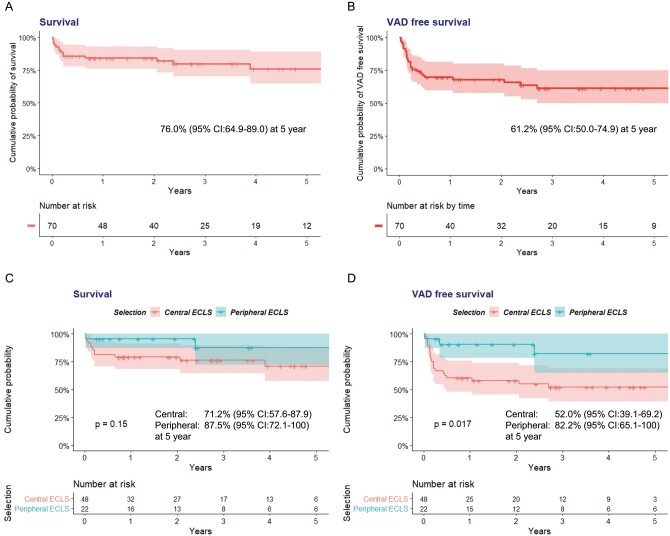
Survival rate and ventricular assist device-free survival rate of (**A** and **B**) total cohort and (**C** and **D**) each support group. CI: confidence interval; ECLS: extracorporeal life support; VAD: ventricular assist device.

The 5-year cumulative probability of survival and VAD-free survival in the total cohort was 76.0% [95% confidence interval (CI) 64.9–89.0] and 61.2% (95% CI 50.0–74.9), respectively (Fig. 3A and B). The 5-year cumulative probability of survival was not significantly different between patients primarily supported by central ECLS (71.2%, 95% CI 57.6–87.9) and peripheral ECLS (87.5%, 95% CI 72.1–100) (*P* = 0.15). The 5-year cumulative probability of VAD-free survival in the central ECLS group was 52.0% (95% CI 39.1–69.2), whereas that in the peripheral ECLS group was 82.2% (95% CI 65.1–100) (*P* = 0.017) (Fig. 3C and D).

One death of unknown cause occurred 872 days after weaning from peripheral ECLS. Weaning from central ECLS was completed within 150 days from the decision point. Four patients died, including 2 with recurrent congestive heart failure at 42 and 682 days after postoperative weaning, 1 with necrotizing pancreatitis at 27 days and 1 with septicaemia at 64 days. Other complications are described in the Supplementary Material.

### Predictive factors affecting functional recovery

Thirty of 48 patients showed functional recovery sufficient for weaning from central ECLS (Table 2). The predictive factors of functional recovery were investigated, and Table 3 shows the results of the univariable logistic regression. In the multivariable logistic regression analysis, a peak CK-MB level of >180 IU/l (adjusted odds ratio 8.61, 95% CI 2.08–35.53; *P* = 0.003), rhythm disturbance (asystole plus CAVB) (adjusted odds ratio 12.3, 95% CI 2.19–69.18; *P* = 0.004) and aortic valve closure at central ECLS conversion surgery (adjusted odds ratio 5.82, 95% CI 1.36–24.98; *P* = 0.018) were negative factors for weaning representing functional recovery (Table 4).

**Table 3:** Predictors of non-myocardial recovery in patients with central ECLS

Variables	Univariate analysis		
	Odds ratio	95% confidence interval	*P*-value
Age (years)	0.53	0.21–1.37	0.193
Body surface area (m^2^)	1.35	0.59–3.06	0.475
Male gender	2.29	0.68–7.70	0.182
Peak CK-MB levels > 180 IU/ml	8.00	2.12–30.15	0.002
Rhythm disturbance (asystole + CAVB)	7.50	1.78–31.62	0.006
Aortic valve closed at central ECLS conversion surgery	4.58	1.22–17.22	0.024
Biventricular support	1.30	0.36–4.68	0.688
Interval between initiation of peripheral ECLS and central ECLS conversion (days)	1.76	0.96–3.22	0.067
Interval of biventricular support (days)	1.08	0.90–1.29	0.396

CAVB: complete atrioventricular block; CK-MB: creatine kinase-MB; ECLS: extracorporeal life support.

**Table 4:** Multivariable logistic regression analysis of predictors of non-myocardial recovery in patients with central ECLS

Variables	Multivariate analysis		
	Adjusted odds ratio	95% confidence interval	*P*-value
Peak CK-MB levels > 180 IU/ml	8.61	2.08–35.53	0.003
Rhythm disturbance (asystole + CAVB)	12.3	2.19–69.18	0.004
Aortic valve closed at central ECLS conversion surgery	5.82	1.36–24.98	0.018

Analysis was adjusted for age and sex.

CAVB: complete atrioventricular block; CK-MB: creatine kinase-MB; ECLS: extracorporeal life support.

## DISCUSSION

FM requires optimum ECLS until myocardial inflammation resolves. Although first-choice treatment is peripheral ECLS, including the Impella, we aggressively upgraded from peripheral to central ECLS in patients with suboptimum ECLS. However, we do not consider that all patients treated by peripheral ECLS would benefit from conversion to central ECLS. The benefits of conversion would be overwhelmed by potential complications related to the conversion surgery in patients with relatively preserved cardiac function, in whom cardiac function would promptly and fully recover under peripheral ECLS only. Conversely, direct LV unloading by central ECLS may inhibit the progression of myocardial inflammation in severe FM, in which LV function would be permanently impaired without LV unloading.

We established several types of central ECLS in our study. Selection of the type of central ECLS was determined by RV function and the degree of pulmonary oedema [4, 10]. Patients who underwent conversion to central ECLS more frequently had higher mortality and major in-hospital complications. However, survival after weaning from mechanical support or durable LVAD implantation was not significantly different between patients with peripheral and central ECLS. Therefore, central ECLS has comparable survival to peripheral ECLS despite a more critical pathology of FM.

Our in-hospital mortality rate was lower than that in previous reports [11]. Cardiac function after weaning from ECLS was variable in this study. Most patients were free from cardiac failure, whereas 11 of 47 showed reduced LV function (ejection fraction of <40%) and were treated by medications. Factors affecting cardiac function after weaning from ECLS are modifiable by improving medical and surgical approaches for FM. Prompt initiation of peripheral ECLS and conversion to central ECLS effectively unload the heart by potentially inhibiting progression and expansion of myocardial inflammation [12–14].

Peak CK-MB, rhythm disturbance and aortic valve closure represent severe myocardial damage by FM. Rhythm disturbances, such as CAVB in FM, are explained by inflammation in the conduction pathway [15–17]. In our study, 15 (83%) of 18 non-weaned patients showed CAVB before conversion surgery. However, all but one of these patients showed recovery to sinus rhythm despite reduced cardiac function. This suggests that CAVB in FM does not represent irreversible damage in conduction systems. Conversely, the most critical form of rhythm disturbance in FM is cardiac arrest. However, 5 of the 7 patients with asystole in our study recovered spontaneous heart beats. Two had no cardiac electrical activity thereafter. This indicates that rhythm disturbances, including asystole, are reversible depending upon the degree of myocardial inflammation and damage. The effect of conversion surgery on myocardial damage or recovery was unclear in our study. However, we consider that reduced LV wall stress by conversion surgery contributed to the relief of LV inflammation and consequently preservation of LV function.

We removed the IABP once central ECLS was established. IABP support enhances opening of the aortic valve, which inhibits thrombus formation in the aortic root and preserves valve function. However, central ECLS, in which outflow cannulation is located in the ascending aorta, effectively washes out the aortic root. In addition, central ECLS is used as a temporary bridge to recovery or to durable LVAD and does not damage aortic valve function.

The Impella, which is one choice for peripheral ECLS, results in LV unloading. This is not feasible by other devices, such as VA-ECMO or the IABP. Combination of the Impella and VA-ECMO, termed ECPELLA, achieves LV unloading and end-organ perfusion [18, 19]. However, we do not consider that ECPELLA fully replaces central ECLS for patients who have FM with a critical haemodynamic state. Support by ECPELLA is not as powerful as that by extracorporeal LVAD in terms of support flow and LV volume/pressure reduction. In addition, prolonged use of ECPELLA increases the risk of leg complications and, more importantly, aortic valve insufficiency. Impella-induced aortic valve insufficiency impairs post-weaning outcomes of patients with reduced LV function. Therefore, we consider that central ECLS is more beneficial than the Impella or ECPELLA in patients requiring prolonged full-flow support. Conversely, the Impella should be indicated in patients who develop mild lung oedema under peripheral ECLS or in those whose functional prognosis is unpredictable.

Histological findings are important to predict the viability of cardiac tissue and determine the mode of mechanical support. The degree of disarray of myocardial tissue and accumulation of inflammatory cells represent the viability of cardiac tissue. In addition, the phenotype of inflammatory cells is a major determinant of the prognosis of cardiac tissue in FM. Development of quantitative assessment in histological studies is warranted.

This study was limited by its retrospective, single-centre design and small sample size Because of the rarity and advanced stage of FM, an in-depth review of each case and/or meta-analysis would improve our understanding of this disease. In addition, because the pump catheter programme was launched in January 2018, selection of ECLS was different before and after this point.

## CONCLUSION

In conclusion, survival after weaning from mechanical support or after durable LVAD implantation is not significantly different between patients with peripheral and central ECLS. Therefore, central ECLS is feasible and safe. Patients with severe myocardial injury as shown by high CK-MB levels, rhythm disturbance and aortic valve closure should be converted to a durable LVAD.

## SUPPLEMENTARY MATERIAL

Supplementary material is available at *EJCTS* online.

## Supplementary Material

ezab231_Supplementary_Data

## Notes

Presented at the 34th Annual Meeting of the European Association for Cardio-Thoracic Surgery, Barcelona, Spain, 8–10 October 2020.

## ABBREVIATIONS

CAVB: Complete atrioventricular block
CI: Confidence interval
CK: Creatine kinase
ECLS: Extracorporeal life support
ECMO: Extracorporeal membrane oxygenation
FM: Fulminant myocarditis
IABP: Intra-aortic balloon pump
IQR: Interquartile range
LV: Left ventricular
LVAD: Left ventricular assist device
RV: Right ventricular
VAD: Ventricular assist device
VA-ECMO: Venoarterial extracorporeal membrane oxygenation
