# Supplementary material for: Efficacy of central extracorporeal life support for patients with fulminant myocarditis and cardiogenic shock
Source: Eur J Cardiothorac Surg. 2021 Jun 25;60(5):1184–92. doi: 10.1093/ejcts/ezab231 (PMC8562956; doi:10.1093/ejcts/ezab231)
Supplement: ezab231_Supplementary_Data [file ezab231_supplementary_data.docx]

**Supplementary material**

**Supplementary methods**

***Diagnosis and Definition of Fulminant Myocarditis***

All patients were diagnosed with FM on the basis of the following findings: 1) a history of infection within 4 weeks; signs of inflammation based on a high fever (>38°C) with an increased white blood cell count and C-reactive protein levels; 2) angiographically normal coronary artery anatomy; 3) evidence of myocardial damage defined as considerable changes in electrocardiographic and echocardiographic parameters, and elevation in serum CK-MB levels; 4) sudden and refractory cardiogenic shock, cardiac arrest, or severe haemodynamic instability, despite aggressive inotropic drugs and those who required peripheral ECLS [1, 2]; and 5) histological findings of myocardial inflammation.

***Selection of the Extracorporeal Life Support System***

There were two types of ECMO system used. 1) The Endumo system was composed of a centrifugal blood pump (Rota Flow; Maquet, Rastatt, Germany) and an oxygenator (Biocube; Nipro, Osaka, Japan). 2) The CAPIOX system was also composed of a centrifugal blood pump (CAPIOX SL Pump; Terumo, Tokyo, Japan) and an oxygenator (CAPIOX-LX, Terumo).

Additionally, there were two types of pumps used in central ECLS, including a pulsatile pump (Nipro VAD; Nipro) and a centrifugal pump (Biofloat; Nipro).

The standard modality of central ECLS was LVAD; however, fulminant myocarditis is often associated with RV failure and lung oedema, for which RVAD and/or ECMO are required.we selected one LVAD system (Type 1), three temporary biventricular support systems (Types 2–4), or one conventional biventricular support system (Type 5) depending on the patient’s condition (figure 2).

Type 1 was an LVAD with LV inflow and aortic outflow (n=15).

Type 2 was an LVAD and RA drainage (LVAD-ECMO) with an oxygenator (n=6).

Type 3 was temporary bilateral VADs (tBiVAD) by an LVAD and a temporary right ventricular assist device (RVAD) with right atrial (RA) inflow and pulmonary artery (PA) outflow (n=23).

Type 4 was central ECMO with RA inflow and aortic outflow with left atrial (LA) drainage (n=3). Type 5 was BiVAD by an LVAD and an RVAD with right ventricular (RV) inflow and PA outflow (n=1) [3].Type 2 LVAD-ECMO achieved LV unloading with reduction of pulmonary blood flow by RA inflow, while type 4 central ECMO was selected in patients with severe coagulopathic oedema of the LV and the thoracic organs. In Types 2–4, once RV function and pulmonary oedema sufficiently recovered, cannulation to the right heart was removed to establishan LVAD (Type 1). Patients who failed to show recovery of RV function underwent conversion surgery to the BiVAD Type 5 system.

The Nipro pulsatile pump (Nipro VAD; Nipro, Osaka, Japan) was the first choice for the LVAD, but in patients with a large body surface area or severe end-organ dysfunction, the centrifugal pump (Biofloat; Nipro) was chosen [4]. The details of these systems are described in the Supplementary Material.

The Central ECLS included the following five systems that were dependent on inflow and outflow as follows.

In Type 1, 15 patients had an LVAD, with the Nipro pump in 13 patients and the Biofloat pump in 2 patients.

In Type 2, six patients had an LVAD and RA drainage with an oxygenator. The Biofloat with the Biocube was used for the LVAD in three patients, the Endumo system for the LVAD in one patient, and the CAPIOX system for the LVAD in two patients.

In Type 3, 23 patients had tBiVAD (an LVAD and temporary RVAD by inferior vena cava drainage in 10 patients and by femoral vein drainage in 13 patients). The Nipro pump was used for the LVAD and the Endumo system for the RVAD in 15 patients. The Biofloat was used for both the LVAD and RVAD in one patient. The Nipro pump was used for the LVAD and the CAPIOX system for the RVAD in one patient. The Biofloat was used for the LVAD and the Endumo system for the RVAD in three patients. The Biofloat was used for the LVAD and the CAPIOX system for the RVAD in two patients. The Biofloat with the Biocube was used for the LVAD in one patient.

In Type 4, three patients had central ECMO and LA drainage established by the Endumo system. In Type 5, one patient had BiVAD (an LVAD and RVAD), with the Nipro pump for the LVAD and the Endumo system for the RVAD.

***Protocol of Weaning from Extracorporeal Life Support***

The LVAD weaning protocol was based on the Berlin criteria as follows: left ventricular ejection fraction (LVEF) >45%, left ventricular internal diameter in diastole (LVDd) <55 mm, and pulmonary arterial and central venous pressure in the normal range during the pump off test [5]. Even if the patient failed to meet the Berlin criteria, the LVAD was removed electively in those who had an LVEF >30%, LVDd <65 mm, and no significant decrease in LVEF, as well as no increase in pulmonary capillary wedge pressure during the pump off test [6].

***Endomyocardial Biopsy***

In a microscopic examination, myocarditis was diagnosed by the presence of an inflammatory infiltrate and associated necrosis or damage of myocytes, which are not characteristic of an ischaemic event. Borderline myocarditis was diagnosed by the findings of a less intense inflammatory infiltrate and no light microscopic evidence of destruction of myocytes [2]. As a result, all patients were diagnosed with myocarditis.

**Supplementary results**

In Type 3, of the six patients with an LVAD and RA drainage, the RA drainage cannula was removed to establish the LVAD in three patients. The remaining three patients underwent conversion surgery to BiVAD(Type 5) at 2, 6, and 10 days after the primary surgery.

In Type 3, among 20 patients with tBiVAD by a temporary RVAD, the temporary RVAD was removed to establish an LVAD in only 14 patients at 2–8 days after the primary surgery. The remaining eight of nine patients underwent upgrade surgery to an extracorporeal RVAD (BiVAD Type 5) at 3–17 days after the primary surgery. One patient died under this support.

In Type 4, of the three patients with central ECMO with LA drainage, one underwent conversion surgery to Impella 5.0 implantation.

***Variable Cardiac Function Post-weaning from Central Extracorporeal Life Support***

Cardiac function was echocardiographically assessed at turning point in surviving patients who showed functional recovery (n=47). According to the ejection fraction, patients were assigned into groups of those with normal LV function (LVEF ≥40%, n=36 [76.6%]) and those with reduced LV function (LVEF <40%, n=11 [23.4%]). There was an approximately 10-mm difference in diastolic and systolic dimensions at all points between the two groups (Supplemental Table 2).

***Complications of Each system***

Complications after weaning-off from the Impella included aneurysm formation of the right axillary artery. The Impella was inserted through the vascular tube in one patient who underwent endovascular repair (VIABAH; W. L. Gore, Flagstaff, AZ, USA) at 66 days after weaning from the Impella 5.0. No patients showed moderate or high aortic insufficiency after weaning from the Impella.

Other major complications after weaning-off from the Central ECLS included LV cuff infection in one patient who underwent complete removal of the cuff at 48 days after weaning. Additionally, there was pseudoaneurysm formation of the LV apex in one patient who underwent surgical repair at 629 days after weaning . In patients who failed to show recovery of cardiac function, durable LVAD implantation was performed for the bridge-to-transplantation purpose in 10 patients within 180 days post-primary surgery. Of them, heart transplantation was performed in two patients at 964 and 1187 days after durable LVAD implantation. Two patients died under durable LVAD support related to intracranial haemorrhage and septicaemia, while the remaining six patients were awaiting heart transplantation under durable LVAD support.

Supplemental Table 1. Echocardiography data at turning point and follow-up point

|  |  | Overall  (n=70) | Central  (n=48) | Peripheral  (n=22) |
| --- | --- | --- | --- | --- |
| Turning point | |  |  |  |
|  | IVST, mm | 9.0 [8.0, 10.0] | 9.0 [8.0, 10.0] | 8.0 [7.2, 8.0] |
|  | PWT, mm | 9.0 [7.0, 10.0] | 9.0 [7.0, 10.0] | 8.5 [8.0, 9.0] |
|  | LVDd, mm | 41.0 [37.0, 49.0] | 41.0 [36.5, 47.0] | 53.5 [44.0, 57.7] |
|  | LVDs, mm | 32.0 [27.0, 41.0] | 31.0 [26.5, 40.0] | 42.0 [38.0, 46.0] |
|  | FS, % | 22.0 [13.0, 31.0] | 24.0 [12.7, 32.0] | 20.0 [14.0, 20.0] |
|  | LVEF, % | 41.0 [24.0, 52.0] | 44.0 [25.5, 54.0] | 31.0 [18.0, 38.0] |
| Follow-up point | |  |  |  |
|  | Interval between the turning point and the follow-up point, days | 20 [11 31] | 22 [12, 33] | 17 [8, 29] |
|  | IVST, mm | 8.0 [7.00, 9.00] | 8.0 [6.75, 9.0] | 8.2 [7.1, 9.2] |
|  | PWT, mm | 8.0 [7.00, 9.50] | 8.0 [7.00, 9.0] | 8.0 [8.0, 10.0] |
|  | LVDd, mm | 46.0 [41.0, 50.1] | 44.5 [40.0, 49.2] | 48.0 [44.5, 51.2] |
|  | LVDs, mm | 33.0 [30.0, 40.5] | 32.50 [29.7, 39.0] | 34.5 [31.5, 42.5] |
|  | FS, % | 25.0 [18.0, 31.0] | 23.5 [17.7, 30.5] | 26.0 [19.0, 31.0] |
|  | LVEF, % | 44.2 [33.6, 54.1] | 43.0 [32.9, 53.3] | 45.9 [34.9, 54.1] |

Data are presented as median [interquartile range]. FS, fractional shortening; IVST, interventricular septum thickness; LVDd, left ventricular internal diameter in diastole; LVDs, left ventricular internal diameter in systole; LVEF, left ventricular ejection fraction; PWT, posterior wall thickness.

**Supplemental Table 2.** Characteristics of normal left ventricular function (EF ≥40%) vs reduced left ventricular function (EF <40%) in surviving patients

|  |  |  | | Normal LV function  (n=36) | | Reduced LV function  (n=11) | |  |
| --- | --- | --- | --- | --- | --- | --- | --- | --- |
| Preoperative characteristics | | | |  | |  | |  |
|  | Age, years | |  | | 47 [38, 60] | | 55 [46, 59] | |
|  | Male sex | | | | 14 (38.9) | | 10 (90.9) | |
| Pathology | | | | |  | |  | |
|  | Lymphocytic myocarditis | | | | 26 (72.2) | | 8 (72.7) | |
|  | Eosinophilic myocarditis | | | | 5 (13.9) | | 0 (0.0) | |
|  | Giant cell myocarditis | | | | 0 (0.0) | | 3 (27.3) | |
|  | Borderline myocarditis | | | | 5 (13.9) | | 0 (0.0) | |
| Laboratory data | | | | |  | |  | |
|  | peak CK | | | | 1854 [465, 4276] | | 1682 [832, 1877] | |
|  | peak CK-MB | | | | 100 [35, 147] | | 83 [64, 118] | |
| Electrocardiographic disturbance | | | | |  | |  | |
|  | Complete atrioventricular block | | | | 15 (41.7) | | 8 (72.7) | |
|  | Asystole | | | | 1 (2.8) | | 1 (9.1) | |
| Support selection | | | | |  | |  | |
|  | Central ECLS | | | | 22 (61.1) | | 6 (54.5) | |
|  | LVAD | |  | | 6 (27.3) | | 2 (33.3) | |
| Time course | | | | |  | |  | |
|  | Interval between symptom onset and initiation of peripheral ECLS support | | | | 5 [3, 7] | | 8 [5, 20] | |
|  | Interval between initiation of peripheral ECLS support and central ECLS conversion, days | | | | 1 [1, 2] | | 6 [4, 7] | |
|  | Central ECLS support, days | | | | 12.0 [7.5, 35.5] | | 45.00 [37.7, 62.0] | |
|  | Interval of biventricular support, days | | | | 5.0 [3.5, 7.0] | | 6.0 [5.5, 10.7] | |
| Transthoracic echocardiography | | | | |  | |  | |
|  | LVDd, mm | | at decision | | 45.0 [41.0, 49.0] | | 56.0 [54.5, 57.5] | |
|  |  | | at turning point | | 40.0 [36.0, 43.0] | | 49.0 [46.0, 54.0] | |
|  |  | | at follow-up | | 45.0 [41.0, 47.0] | | 52.0 [50.2, 55.0] | |
|  | LVDs, mm | | at decision | | 39.0 [34.5, 45.0] | | 52.0 [47.5, 55.0] | |
|  |  | | at turning point | | 30.0 [25.5, 33.0] | | 42.0 [39.0, 44.0] | |
|  |  | | at follow-up | | 32.0 [29.0, 34.2] | | 45.0 [41.5, 48.8] | |
|  | LVEF, % | | at decision | | 12.5 [8.3, 23.9] | | 9.8 [7.4, 14.0] | |
|  |  | | at turning point | | 49.0 [34.0, 58.0] | | 35.0 [26.0, 41.50] | |
|  |  | | at follow-up | | 51.7 [45.0, 55.8] | | 31.1 [19.8, 35.7] | |

Data are presented as median [interquartile range] or number (%). CK, creatine kinase; ECLS, extracorporeal life support; LVAD, left ventricular assist device; LVDd, left ventricular internal diameter in diastole; LVDs, left ventricular internal diameter in systole; LVEF, left ventricular ejection fraction; RV, right ventricle.

[1] Pages ON, Aubert S, Combes A, Luyt CE, Pavie A, Léger P *et al.* *Paracorporeal pulsatile biventricular assist device versus extracorporal membrane oxygenation-extracorporal life support in adult fulminant myocarditis*. J Thorac Cardiovasc Surg 2009;**137**:194-7.

[2] Lorusso R, Centofanti P, Gelsomino S, Barili F, Di Mauro M, Orlando P *et al.* *Venoarterial Extracorporeal Membrane Oxygenation for Acute Fulminant Myocarditis in Adult Patients: A 5-Year Multi-Institutional Experience*. Ann Thorac Surg 2016;**101**:919-26.

[3] Fukushima S, Tadokoro N, Koga A, Shimahara Y, Yajima S, Kakuta T *et al.* *Central conversion from peripheral extracorporeal life support for patients with refractory congestive heart failure*. Journal of artificial organs : the official journal of the Japanese Society for Artificial Organs 2020.

[4] Seguchi O, Fujita T, Kitahata N, Iwasaki K, Kuroda K, Nakajima S *et al.* *A Novel Extracorporeal Continuous-Flow Ventricular Assist System for Patients With Advanced Heart Failure　- Initial Clinical Experience*. Circ J 2020;**84**:1090-96.

[5] Dandel M, Weng Y, Siniawski H, Potapov E, Lehmkuhl HB, Hetzer R. *Long-term results in patients with idiopathic dilated cardiomyopathy after weaning from left ventricular assist devices*. Circulation 2005;**112**:I37-45.

[6] Matsumiya G, Saitoh S, Sakata Y, Sawa Y. *Myocardial recovery by mechanical unloading with left ventricular assist system*. Circ J 2009;**73**:1386-92.
